# A Lightweight Authentication MAC Protocol for CR-WSNs

**DOI:** 10.3390/s23042015

**Published:** 2023-02-10

**Authors:** Bashayer Othman Aloufi, Wajdi Alhakami

**Affiliations:** Department of Information Technology, College of Computers and Information Technology, Taif University, P.O. Box 11099, Taif 21944, Saudi Arabia

**Keywords:** cognitive radio network, wireless sensor network, lightweight, authentication, CR-WSNs, MAC

## Abstract

Cognitive radio (CR) has emerged as one of the most investigated techniques in wireless networks. Research is ongoing in terms of this technology and its potential use. This technology relies on making full use of the unused spectrum to solve the problem of the spectrum shortage in wireless networks based on the excessive demand for spectrum use. While the wireless network technology node’s range of applications in various sectors may have security drawbacks and issues leading to deteriorating the network, combining it with CR technology might enhance the network performance and improve its security. In order to enhance the performance of the wireless sensor networks (WSNs), a lightweight authentication medium access control (MAC) protocol for CR-WSNs that is highly compatible with current WSNs is proposed. Burrows–Abadi–Needham (BAN) logic is used to prove that the proposed protocol achieves secure and mutual authentication. The automated verification of internet security protocols and applications (AVISPA) simulation is used to simulate the system security of the proposed protocol and to provide formal verification. The result clearly shows that the proposed protocol is SAFE under the on-the-fly model-checker (OFMC) backend, which means the proposed protocol is immune to passive and active attacks such as man-in-the-middle (MITM) attacks and replay attacks. The performance of the proposed protocol is evaluated and compared with related protocols in terms of the computational cost, which is 0.01184 s. The proposed protocol provides higher security, which makes it more suitable for the CR-WSN environment and ensures its resistance against different types of attacks.

## 1. Introduction

The current wave of wireless sensor network (WSN) research began around 1998 and continues to gain momentum [1]. Furthermore, WSNs have attracted significant interest as a ubiquitous technology. They can be combined with various next-generation technologies [2], and WSNs can support a wide range of real-world applications.

In general, WSNs are specialized wireless networks consisting of a network of wireless sensors, each of which is placed in a specific location to measure environmental parameters, such as temperature, humidity, light, motion, or vibration. The sensors can transmit data to each other via radio using wireless tools [2]. WSNs typically consist of a gateway, a sensor node, and a user, all of which are limited resources in smart devices. Sensors are used in various fields to capture vast amounts of data in real-time. As a result, the portal deals with the data obtained by the sensors to provide services to authorized users [3,4], since the WSNs consist of many sensor nodes with limited capacity for processing, storage, and communication. They are widely used in a variety of fields, including banks, hospitals, research, national defense institutions, etc., [5]. However, valuable services are exposed to security risks, as sensitive data are transmitted through public channels in many categories. As a result, secure authentication mechanisms must provide a wide range of services in a WSN [3]. 

Wireless networks provide benefits in various disciplines, and they continue to be the subject of much research. As a result, new studies are constantly being published in efforts to improve this technology. It is clear that WSNs face several issues and challenges that must be addressed [6]. Therefore, achieving solutions to these problems is an essential part of the research. WSN nodes have limited resources and limited storage capacity, which is one of the most critical difficulties [6,7]. As a result, creating a proper lightweight security mechanism is critical to conserving these resources and providing significantly better performance to avoid slower communication between network entities.

Due to the industrial scientific and medical (ISM) being an open band, many other wireless applications use this band, including wireless fidelity (Wi-Fi), wireless microphones, Bluetooth, microwave ovens, etc., which causes the major problem of the scarcity of the spectrum that comes from the interference between different wireless applications using this band [8]. WSN communications operate in the ISM domain [8]. The ISM band is highly congested because many wireless applications rely on this band to accomplish their communications. Thus, we can say that the scope of ISM covers a wide range of applications and is growing exponentially. Since many of these devices operate on the same unlicensed frequencies as the ISM, they can create many problems in densely populated areas (particularly 2.4 GHz). The presence of a WSN combined with the uncoordinated deployment of other WSNs or devices from other technologies, such as Wi-Fi and Bluetooth, can significantly degrade the service and signal strength due to the general interference levels. Interventions in crowded urban environments may become more common in the future. The evaluation of WSN channel capacity performance in crowded urban situations using the ISM band is critical to mitigating an already severe congestion problem that is worsening [8].

WSNs can be improved by using cognitive technologies to help them overcome the limitations of traditional wireless networks. In traditional wireless networks, incorporating perceptual radio capabilities provides a feasible strategy for delivering high-quality data transmissions. Perceptual radio sensor networks offer a new way to efficiently and opportunistically make the most of the licensed spectrum resources. These features include reduced packet loss, reduced power wastage, and improved connection quality. In short, the CR effectively deals with the increasing demand for efficient channels and the limitations of the wireless spectrum by enabling the CUs to have opportunistic access to the licensed spectrum without interfering with the PUs.

The spectrum can range from 3 kHz to 300 GHz. It is a valuable natural resource that is underutilized [9]. In 1999, Joseph Mitula created the cognitive radio network (CRN) to communicate with other radio stations. It is a technology that allows dynamic spectrum access (DSA) across underexploited bands. Using the white space portions is the vital role of Cognitive Radio (CR) technology that enables the spectrum to be used more efficiently [10,11]. Therefore, cognitive radio networks (CRNs) are a sophisticated and adaptive promising technology that sense licensed channels and identify unused bands that are recognized as a spectrum hole or white space; this technology allows the spectrum to be used more efficiently. CRNs consist of primary users (PUs), also known as licensed users (LUs), who are the authorized users to whom the data channel is assigned, and cognitive users (CU), also known as secondary users (SUs), who use the white space of the spectrum simply to increase the spectrum efficiency and utilization. Protecting the activities of the LUs is necessary because these users have priority access to the authorized channels. Thus, the CUs can enhance spectrum utilization by sensing and identifying white spaces before data transmission begins and leaving these channels occupied as soon as LU activity is detected [12]. A collaborative CRN approach needs a specialized common control channel (CCC) that ensures that control frames are exchanged successfully. Moreover, it simplifies the CU’s communication and collaboration, allowing CUs to share spectrum sensing results and select an authorized channel to transmit the data between the transmitter and the receiver [10,11,13]. Despite the uncomplicated design of the CCC, it solves problems such as resource allocation, establishing links between CUs, and monitoring secure communication [14].

### 1.1. Functions of the CRN Core

**Figure 1:**
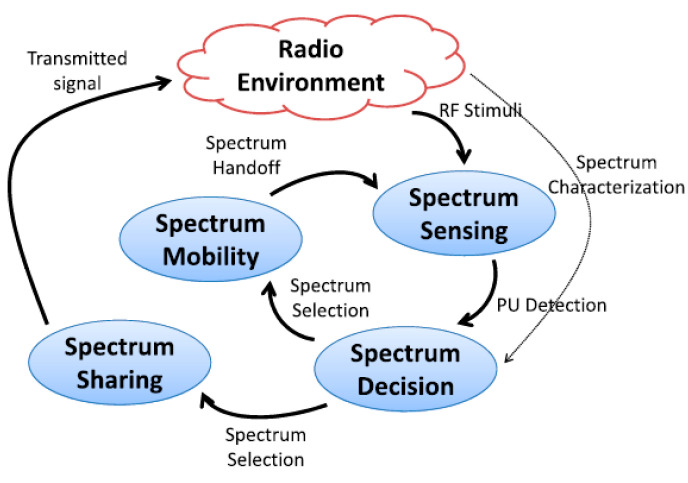
The cognitive cycle implemented by each CR node [15].

To perform their functionalities for CU communications, CRNs have four fundamental functions linked to each other as follows and as illustrated in Figure 1:

- Spectrum sensing;
- Spectrum decision;
- Spectrum sharing;
- Spectrum mobility.

The television (TV) band is used to integrate the CRN and WSN networks, primarily used to transmit data on a channel other than the control message channel. If we suppose all these applications use WSN, Wi-Fi, and Bluetooth in the same ISM band, then there is the potential for sudden amounts of interference, reduced WSN channel capacity, and decreased performance of the WSN and other wireless systems [8]. Most of the licensed spectrum is untapped. Therefore, sharing unlicensed idle bandwidth between unlicensed devices is a viable solution to the problem of spectrum scarcity. Sensor nodes (as SUs) can detect and use the unused licensed spectrum when the primary users (PUs) are not using it [16].

Radio (CR-WSN) is a type of WSN consisting of sensor nodes with cognitive capabilities. These CR nodes are placed across the network to monitor physical or environmental conditions jointly. CR-WSNs must take advantage of the available spectrum holes or white spaces to function. The use of television white space, which has a higher spectrum efficiency than analog television, has resulted in the emergence of more television white space bands, as the changing trends from analog to digital television have led to the release of more channels in television white space bands. Sharing the spectrum across the TV white space spectrum is important because it is the first step towards its effective and dynamic use through the CR [17].

### 1.2. Contributions of the Proposed Protocol

The aim of this research is to develop lightweight secure cognitive radio-enabled wireless sensor networks by integrating both WSN and CRN technologies for improving channel utilization and network performance. This can be achieved by adopting hash algorithms (SHA-1) and XOR operations leading to a lower computational cost as compared to other conventional security algorithms. The contributions of the proposed protocol can be summarized as follows:

- To overcome the challenges in WSNs by using CRNs to provide available channels to the network, leading to a significant improvement in the performance of data exchange by ensuring on-time data delivery without any delay, due to sending the data in a specific channel number in the TV band, which is considered to be less congested than the ISM band in WSNs. Thus, we ensure that the network is not degraded through the integration process of the WSNs and CRNs.
- The critical challenges in WSNs are that the WSN nodes have limited power resources and limited storage capabilities. Thus, finding a suitable lightweight security mechanism is necessary to maintain these resources and obtain better results by designing an approach depending on a lightweight process to achieve mutual authentication in order to enhance the security of the WSN environment relying on the concept of the CRNs. To conserve these resources, lightweight algorithms were used in the proposed protocol.
- The proposed protocol has a powerful and impenetrable defense against many related attacks, for instance, a session-specific random number leakage attack, a privileged insider attack, a stolen smart card attack, a replay attack, a man-in-the-middle (MITM) attack, a sensor node capture attack, an offline password guessing attack, a key compromise impersonation, a stolen verifier attack, and an insider attack. In addition, we demonstrate the mutual authentication and the perfect forward secrecy of the proposed protocol.

There are five sections in this paper. Section 2 reviews the recent related literature, focusing on how other researchers have used lightweight algorithms to provide the authentication aspect of WSNs. In Section 3, the proposed MAC protocol and its design process are described. The implementation of the Burrows–Abadi–Needham (BAN) logic and the automating of the validation of internet security protocols and applications (AVISPA) security validation tool are discussed in Section 4. Section 5 discusses and evaluates the implementation of the protocol. Finally, Section 6 concludes the paper and offers suggestions for future research.

## 2. Literature Review

This section covers the methods that other researchers have proposed in the past five years to contribute to a secure wireless networking environment. There are significant challenges facing the WSN, including the unavailability of the spectrum for wireless devices in the network due to the increase in the number of wireless applications, which leads to split channels and spectrum shortage, the most common problems of this network. Therefore, a CRN can be used to work around this problem. Due to the lack of available resources providing integrated security for CR-WSNs, it is necessary to study and investigate modern security mechanisms used to secure both WSNs and CRNs. It is important to highlight the most recently published work in each field and understand the proposed security operations.

### Security Protocols for CRNs and WSNs

This section presents the security protocols implemented in wireless sensor networks. In addition, the lightweight protocols executed in both CRNs and WSNs are reviewed to ensure the security of these environments.

For instance, in [18], data-driven CRNs were implemented, and the SUs had to submit information to the database to receive information regarding the channel allocation process. To address this issue, the authors in [18] developed a privacy-preserving lightweight technique for secondary users in data-driven CRNs. The system used XOR, hash functions, and symmetric cryptography for channel validation and allocation. The security analysis exploited various vulnerabilities, including method shields against impersonation, modification, replay, and eavesdropping assaults, while also providing identity and location concealment, reciprocity authentication, and resistance to multiple threats. Moreover, their scheme outperformed the existing schemes in terms of computation, transmission cost, and delay, according to the performance investigation results.

In [4], Kwon et al. demonstrated that Moghadam et al.’s approach was susceptible to insider attacks and session-specific leaks of random numbers. Moghadam and colleagues described the method comprising each entity generating a session key by multiplying the elliptic curve cryptographic (ECC) function with itself. However, the ECC involves a great deal of computation. If the sensor nodes have limited computing power and storage capacity, the ECC will not provide the real-time communication required by the WSNs. Kwon et al. claimed to have developed a secure, lightweight, and efficient mutual authentication protocol used in wireless sensor networks. Their protocol used hash functions and XOR operations to achieve adequate security while remaining cost-effective. The authors of the paper claimed that their method overcame the security flaws inherent in the scheme developed by Moghadam et al. Consequently, WSN-SLAP (Secure and Lightweight Mutual Authentication Protocol) had good resistance to various attacks, such as the theft of a smart card, an insider stealing the verifier’s ID, an offline password guessing attack, and an episode that introduced a session-specific random number leak. Since WSN-SLAP was equipped with advanced security features, its security and productivity were better than previous comparable technologies, making it an excellent solution for implementing WSNs.

In [19], according to Yu and Park, Mo and Chen’s approach had several security flaws, including potential session key leaks and disguised attacks. Furthermore, there was no guarantee of anonymity, intractability, or mutual authentication. Moreover, their method did not provide non-compliance. Therefore, Yu and Park proposed a lightweight and secure three-factor authentication system using an SLUA-WSN (Secure and Lightweight Three-Factor-Based User Authentication Protocol) that protected against vulnerabilities in its approach. The SLUA-WSN provided users with anonymity, impossibility, and mutual authentication protection from security threats. Concerning the existing authentication protocols, the SLUA-WSN offered a variety of benefits, including preventing node capture and replay attacks, insider attacks, and masquerade attacks, secure intent, and the anonymous authentication of users. The protocol used fuzzy extraction to extend the binary security provided by the SLUA-WSN. The safety and efficiency outperformed previous designs, making them more suitable for practical WSN applications.

In [20], Mo and Chen developed a lightweight three-factor authentication system for WSNs, which was a more secure implementation of their system. To compensate for the previous flaws, an article recently published by Lu et al. examined the previous solution’s security vulnerabilities and offered a three-factor authentication key agreement technique (GAT) for WSNs, a unique approach. The method of authentication applied by their protocol was vulnerable to several security vulnerabilities, such as an offline password-guessing attack, known session-specific temporary information exploits, and no backward security for sessions. To address the limitations of their system, they developed a more robust three-factor authentication system that applied critical agreement. Meanwhile, in a WSN setting, this approach employed ECC without bistatic pair-up to save time while retaining security. Redirection, user registration, logins, authentications, and password resets were the four phases of the approach. Comparing this technique to related ones, it balanced security and efficiency regarding the computing costs and communication overhead. This approach was a feasible lightweight solution for authentication in the WSN setting.

In [21], there was a proposal from Moghadam and others to establish a system based on an elliptic curve Diffie–Hellman mutual authentication and key negotiation. During the investigation, this paper studied the MAJED ALOTAIBI symmetric encrypted system and the biometric-based anonymous user authentication and key agreement protocols. Several issues with the security of the MAJED ALOTAIBI system were found, including stolen verification attacks, impersonation attacks, and issues related to its session key. To overcome the restrictions and establish a secure connection with authorized entities, they proposed using elliptic curve Diffie–Hellman (ECDH)-based authentication and fundamental agreement mechanisms for the WSN infrastructure. A new method of dynamic node addition was offered for the setting of wireless sensor networks. This method generated unique symmetric keys and session keys for each session using a robust key generation algorithm. This article discussed the ECDH approach and showed that the protocol exhibited confidentiality protection, mutual authentication, key agreement, and message integrity. This method was more efficient than comparable techniques and was also equipped with enhanced security mechanisms.

In [22], they proposed a two-level authentication mechanism during communication within a CRN. Security credentials were obtained from an authorized point before the CR nodes joined the network. Public-key and symmetric-key encryption were used in the proposed approach. Instead of other networks that only establish and provide access to licensed users, they authenticated users who did not have authorization to access the network resources. The authors suggested a two-level authentication mechanism to validate the cognitive node and its users. The system displayed a node user using the application layer, data interface, and network levels (physical and logical). Moreover, they argued that the proposed authentication method could protect from reflection attacks, denial-of-service attacks, and man-in-the-middle attacks by working across many layers, making it a highly effective and secure authentication method. Utilizing public-key cryptography and symmetric key cryptography, this approach reduced the cryptographic operations and time required for authentication. Cryptographies based on public-key infrastructure and those based on symmetric keys are used differently. The authentication scheme suggested in this article reduced the number of cryptographic operations and the time necessary to complete the authentication process compared to other methodologies. Based on an analysis conducted by BAN logic and the Scyther verification tool, the suggested approach was confirmed to be correct. There was no success against their authentication methodology for several types of attacks.

In [23], Parvin et al. described a trust-based authentication technique for secure communication in CRNs to guarantee specific interactions. This secure authentication eliminated the security overhead and communication costs. Therefore, this paper proposed an authentication technique based on the CRN concept and based on trust. To ensure network security, the PUs who wished to use the SU’s proposed paradigm had to first verify the SU’s trust value before using the SU’s free spectrum. All the CR nodes stored their trust values in a common Certificate Authority (CA) trusted repository. The trusted repository contained two types of information, one of which was the public value, which all nodes in the network may view. The CA’s third secret was a private value that only the CA could access. The personal trust value was a secret number that was not shown to any other entity (the CA). This value was kept for security reasons. A hacker or attacker could compromise the network and modify the trust value on purpose, but the network could identify which node was compromised, since the CA knew the private trust value. The CA notified all the other nodes in the network when a node was compromised and demanded the deletion. It established secure communications by validating a trust value in a reputable repository. Since this study failed to account for the bias among other nodes, the trust values of all nodes would always be higher.

A novel method of authenticating primary users’ signals in CRNs was presented in [24], which complied with the FCC’s standards for verifying prior users’ signatures. Using these signatures, the solution detected core users when attacks were detected (due to the physical radio channel characteristics). A crucial element for the success of the technique was the physical proximity of the assistance nodes. Using the cryptographic signatures contained in the helper node’s transmissions, the signals from the primary user were checked using the helper node’s movements. A new authentication method was developed for the helper node using physical layer authentication to verify the primary user’s signals. Since the approach was based on the geographic proximity to the primary user, no training was necessary, as it looked at the assistance node’s proximity to the primary user. They investigated the proposed approach by conducting theoretical analysis, experimental evaluation of the CRAWDAD data set, and the design and evaluation of a prototype based on GNUradio. Based on this study, they concluded that primary users’ signals may be utilized in CRNs.

In [25], a digital signature algorithm recognized as an elliptic curve digital signature algorithm (ECDSA) based on spectrum-aware cryptography (SAC) security was proposed in the cognitive radio sensor network (CRSN). The SAC technology uses micro switches to encrypt data to reduce packet delay and power consumption while maximizing the quality of service (QoS). This algorithm determined whether a node was a valid user or a malicious one in two main ways. The first determined whether the node belonged to a valid user. In addition, since the base station (BS) tracked the node IDs and signatures in the session key list, a second illegal node could be detected by multiple key requests from the same BS node identifier. A validation timer was activated after the BS generated the session key list, requiring all nodes to create a key for the response before the timer expired. If the BS received a response from the node before the timer ended, it was deemed malicious. Otherwise, the BS flagged the node as dangerous. The malicious node was removed from the transmission path of all the mechanisms of node authentication. The simulation results indicated that the SAC’s reduced packet latency and power consumption were significantly better than other CRSN protocols.

In [26], to defend against spectrum sensing data falsification (SSDF) attacks, the authors proposed a collaborative spectrum secure (CSS) sensing technology based on the mechanism’s reputation for wireless cognitive sensor networks. The CSS devised a dynamic confidence assessment methodology that identified the reputation value for perceptual sensor nodes based on their past sensor activities by utilizing a beta reputation model. For the fusion decision, the fusion center (FC) assessed the feedback provided in the final fusion decision to determine a fair weight value, which improved the accuracy of the sensing system. The simulations demonstrated that the proposed method could distinguish between reports from honest system units and reports from attackers, which permitted the efficient detection of attackers. This method achieved better sensing performance than previous methods. The proposed solution was suitable for security systems because of its effectiveness in detecting and isolating attackers, in addition to avoiding misunderstandings between honest sensor nodes and those loyal to the attackers. 

Table 1 shows the comparison of the recent approaches to secure CRNs and WSNs.

## 3. Design of the Proposed Lightweight Authentication MAC Protocol for CR-WSNs

This section focuses on the proposed protocol’s design, explains the proposed framework and what security mechanisms were applied in our protocol, and illustrates the proposed protocol’s sequential messages, which consist of four phases.

### 3.1. Architecture of the Proposed Framework

**Figure 2:**
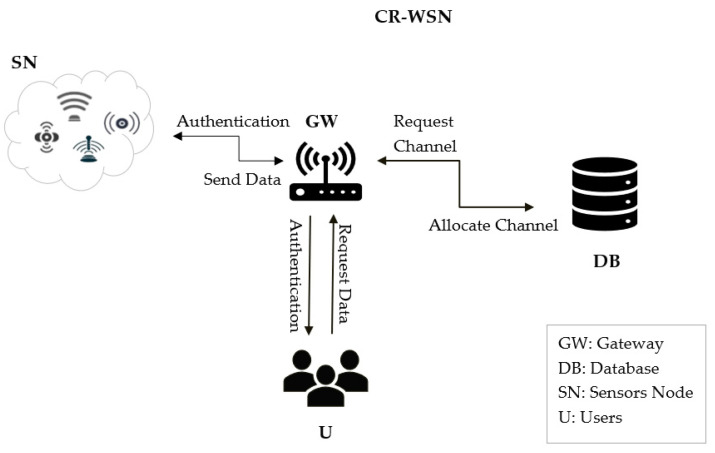
The framework of the proposed lightweight authentication MAC protocol for CR-WSNs.

The design of our proposed protocol depended on developing lightweight secure cognitive radio-enabled wireless sensor networks by integrating both WSN and CRN technologies. The integration process is shown in Figure 2. The model consisted of users Us, a gateway (GW), a sensor node (SN) as a cognitive user (CU), and a database (DB). Therefore, the GW provided a security mechanism, such as a shared key for the sensor and user over a secure channel. Then, the GW authenticated both the sensor and user. Moreover, it communicated with the DB to allocate the channels and provide them for the sensors and the users. The sensor collected the data and sent them to the GW. After that, the user could access the gateway to monitor the sensed data. The secure MAC protocol in the CR-WSNs using a lightweight authentication protocol comprised four phases: the registration phase, consisting of the sensor node registration phase and user registration phase, the login and authentication phase, the control channel exchange phase, and the data exchange phase. The framework helped to technically model the functional requirements of the system by remodeling them into understandable diagrams. We next outline the system model, consisting of the model protocol components and users. Figure 2 shows the framework of the proposed protocol, as follows:

- U: the user obtains the sensed data from the gateway that was collected from the sensors.
- GW: the gateway is responsible for key generation and for prerequesting channels from the DB and allocating channels to sensors and users, as well as the authenticating process, which is conducted mutually, where every entity in the network can authenticate each other.
- SN: the sensor node obtains some secret parameters for authentication and collects data.
- DB: the database is an entity that provides a list of available TV channels.

### 3.2. Security Mechanism Associated with the Proposed Protocol

Two security algorithms were employed in this approach to achieve a range of security and performance goals. Following are brief descriptions of the hash algorithm and the ECC.

#### 3.2.1. Hash Function

Computer science has long used the concept of a “hash function”, which offers a method for transforming a string of an arbitrarily long input text into a fixed-length output string. Although there are several hash functions with various names, those that can be used for cryptographic applications are cryptographic hash functions [27]; also known as hashing algorithms, they are among the essential cryptography tools and may be used for various security purposes, such as authentication, signatures, steganography, and digital timestamping [27]. Ram claims that the use of cryptographic hash functions satisfies different security objectives in various information processing applications.

#### 3.2.2. ECC

In the mid-1980s, both Koblitz and Miller individually introduced the ECC algorithm, which is a public-key cryptography based on public and private keys for both authentication and encryption procedures [28]. In addition to the ECC public key cryptography algorithm’s advantageous characteristics, such as its small key size, fast performance, and lower energy consumption, it has strong security [29]. To implement the ECC in a lightweight authentication MAC protocol for CR-WSNs, we needed to define the parameters used in the process and how these parameters could be calculated to serve the operation in our protocol. E (a, b) represents the elliptic curve, and Q is the base point. Furthermore, these parameters were originally transposed from the Diffie–Hellman cryptography to make use of the discrete logarithm problem [29]; to use it on a protocol efficiently, the D–H’s technique was transposed into the ECC in such a way as to aid the implementation of the algorithm on the proposed protocol.

### 3.3. Proposed Protocol Sequential Messages

This section includes the proposed design of the lightweight authentication MAC protocol for CR-WSNs with four phases described in detail, which are the registration phase consisting of the sensor node registration phase and user registration phase, the login and authentication phase, the control channel exchange phase, and the data exchange phase. Table 2 presents the notations of the proposed lightweight authentication MAC protocol for CR-WSNs.

#### 3.3.1. Registration Phase

This section focuses on the registration phase of the proposed protocol, which consists of the first sensor node registration phase and the user registration. This phase illustrates how the entities registered themselves in the network, as shown in Table 3 and Table 4, respectively.

1. Sensor Node Registration

Step 1: The GSK is defined as a pre-shared key between the gateway and the sensor. The sensor begins by generating a random number Rs and selects its ID_Si_. Next, the sensor computes three different hashes: H1 = h(ID_Si_), H2 = h (ID_Si_ || Rs), and H3 = H1 ⊕ H2; then, it sends {ID_Si_, {H2, H3} _GSK_} to the GW.

Step 2: The GWS is the secret information of the GW. The GW receives and decrypts {ID_Si_, {{H2, H3} _GSK_} ^GSK^}. The GW needs to validate the H3; so, it generates a hash H*1 = h(ID_Si_). Then, it computes H*3 =H*1 ⊕ H2 and compares H3 =? H*3. If valid, it generates a sensor master key, Ki = h (h (ID_Si_, Rs) || GWS). After that, the GW generates a hash, H4 = h (Ki || ID_Si_) ⊕ H2. Then, it links the ID_Si_ with the Ki sensor master key, stores {ID_Si_, H2, Ki} in the local database, and sends this message to the sensor {{Ki}_GSK_, H4}.

**Figure 3:**
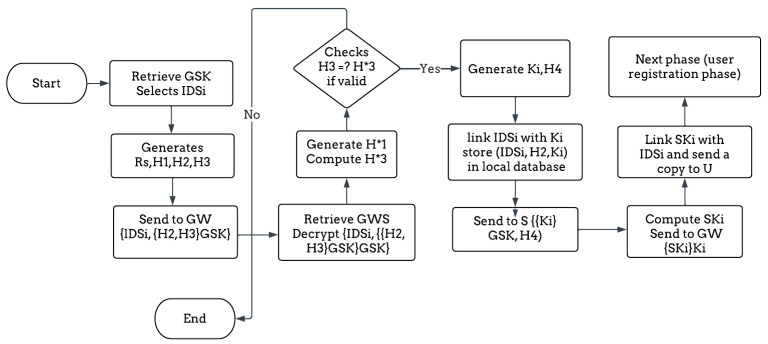
The flow diagram of the sensor registration phase.

Step 3: The S receives and decrypts {{{Ki}_GSK_, H4} ^GSK^}. After receiving the master key from the GW, the sensor calculates SKi = h (H2 || Ki) ⊕ h (Rs) and sends it to the GW encrypted by the Ki to be used later in the data exchange phase. For further details, Figure 3 summarizes the whole process of the sensor registration phase.

2. User Registration

Step 1: Here, both U and GW can generate a shared key by selecting the Upk and computing the user’s public key Upub = Upk * Q. Then, two random numbers RUU and R_i_ are generated. After that, the user computes a hash value HU = h (RUU || Upk) and computes RU = HU * Q. The user computes KU = Upk * ID_G_ to compute the certificate, which contains the IDs of both the user and the gateway, CUS = h (IDi || ID_G_ || RU || KU); then, it sends it to the GW.

Step 2: The GWS is the secret information of the gateway, and the GW generates a random number A, R_g_. The GW computes HID_i_ = h (ID_i_ ∥ R_g_) and PIDi = HIDi ⊕ h(A ∥ GWS). To complete the process of the ECC, the GW selects GWpk and computes GWpub = GWpk * Q. Then, it generates a random number RGG, computes a hash value HG = h (RGG || GWpk), and computes RGW = HG * Q. Finally, it computes KG = GWpk * IDi to compute the CGS = h (ID_G_ || IDi || RGW || KG) and sends it to the U.

**Figure 4:**
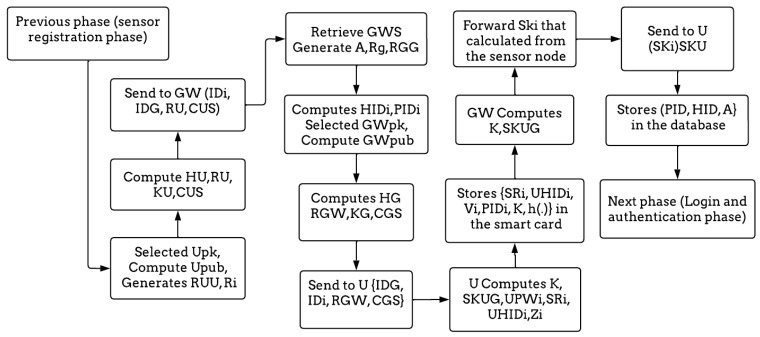
The flow diagram of the user registration phase.

Step 3: Now, both the user and the gateway can calculate the shared key SKUG, which is derived from the ECC. The GW forwards the session key to the user to use when encrypting data. On the other hand, the user computes UPWi = h (PWi ∥ Ri), SRi = Ri ⊕ (IDi ∥ PWi), UHIDi = HIDi ⊕ h (PWi ∥ IDi ∥ Ri), and Zi = h (UPWi ∥ IDi ∥ Ri) and then stores {SRi, UHIDi, Vi, PIDi, K, h(.)} in the smart card. For further details, Figure 4 summarizes the whole process of the user registration phase.

#### 3.3.2. Login and Authentication Phase

This phase shows how the entities log in and authenticate each other based on obtaining the same shared key, as shown in Table 5.

Step 1: After entering the smart card, the user enters the identity IDi and the password PWi. These values are calculated by the smart card, R*1= SRi ⨁ h(IDi||PWi), UPW*i = h (PWi|| Ri), and Z *i =h (UPW*i || IDi|| R*1). After that, the smart card validates the Zi by comparing it with the Zi, which is stored in the smart card. If the validation of Zi is approved, the smart card engenders a random nonce N1 and calculates HIDi = UHIDi ⨁ h(PWi||IDi||Ri), Si = IDSi ⨁ h(PIDi||HIDi), M1 = N1 ⨁ h(HIDi||PIDi), and Z1 = h (IDSi ||PIDi||N1||HIDi). Eventually, the user transmits {PIDi, Si, M1, Z1} to the GW through a public channel.

Step 2: Immediately upon the gateway receiving {PIDi, Si, M1, Z1} from the user, the gateway recaptures the shared secret value A and PIDi from its database. Next, the gateway calculates HID*i = PIDi ⨁h (A || KGW), IDSi * = Si ⨁h (PIDi || HID*i), N*1 = M1 ⨁h (HID *i || PIDi), and Z*1 = h (IDSi * || PIDi|| N*1 || HID*i) and confirms whether Z*1 matches with Z1. When the match is corroborated, the gateway fetches IDSi and h (IDSi || Rj) from its database. The gateway calculates Ki = h (H2 || KGW), M2 = h (N2 || HIDi) ⨁ h(Ki || PIDi), M3 =N1 ⨁h(h(N2 || HIDi) || Ki), and Z2 = h (PIDi || IDSi || h(N2 || HIDi) || N1). Eventually, the GW transmits {PIDi, M2, M3, Z2} to the Si through a public channel.

Step 3: If the sensor received this message {PIDi, M2, M3, Z2}, then the sensor calculates h(N2 || HIDi)* = M2 ⨁ h(A || PIDi), N *1 = M3 ⨁ h(h(N2 || HIDi)* || PIDi), and Z*2 = h(PIDi || IDSi || h(N2||HIDi) || N*1) and investigates whether Z*2 matches Z2. When the match is validated, the Si calculates the SSK = h (h(N2 || HIDi) || N3 || N1), M4 = N3 ⨁ h(Ki||N2), and Z3 = h(SSK ||N3 || IDSi), where the SSK is the session key. Eventually, the sensor transmits {M4, V3} to the gateway.

Step 4: When the gateway receives this message {M4, Z3} from the sensor, the gateway calculates N * 3 =M4 ⨁ h (Ki || N2), SSK * = h(h(N2 || HIDi) ||N * 3 ||N1), and Z*3 = h(SSK* || N*3|| IDSi) and investigates whether the Z*3 and Z3 are equal. When validated, it calculates xnew = h (x || N4), PID newi = HIDi ⨁ h(x new || kGW), Pi = PIDnewi ⨁ h(N3 || HIDi), M5 = N4 ⨁ h(HIDi || IDSi || N3), M6= N3 ⨁ h(N2 ||HIDi || PIDnewi), and Z4 = h(N2 || N3 || PID new i || SSK). Then, the gateway transmits {Pi, M5, M6, Z4} to the user and updates {PIDi, x} to {PIDnewi, xnew}, when the key agreement is completed successfully.

Step 5: When the user receives and decrypts the message {Pi, M5, M6, Z4} from the gateway, the user calculates PIDnewi= Pi ⨁ h(N1 || HIDi), N*2 = M5 ⨁ h(HIDi || IDSi ||N1), N*3 = M6 ⨁ h(N*2 || HIDi || PIDnewi), SSK* = h(h(N*2 || HIDi)||N*3 || N1), and V*4 = h(N*2 || N*3 || PIDnewi || SSK*) and investigates whether Z*4 matches Z4. When the match is confirmed, the user substitutes {PIDi} to {PIDnewi} in the smart card.

Note: Password Update

When a user is required to update the password, the user follows these steps:

Step 1: After entering the smart card, the user enters the identity IDi and the password PWi. The smart card calculates R *i = SRi ⊕ h(IDi\\PWi), UPW *i = h(PWi\\Ri), and Z*i = h(UPWi\\IDi\\R *i ) and verifies the equality of Z *i and Zi. If the verification process is successful, the smart card requests a new password from the user.

Step 2: Ui inputs a new password PW^new^_i_. The smart card selects a random number

R *_i_ and computes UPW^new^_i_ = h(PW^new^_i_\\R ^new^i), SR^new^_i_= R ^new^_i_ ⊕  (IDi\\PW ^new^_i_), UHID ^new^i = HIDi ⊕ h(PW ^new^_i_\\IDi\\R^new^i), and V^new^_i_ = h(UPW^new^_i_\\IDi\\R^new^_i_). Eventually, the smart card stores {SR^new^_i_, UHID^new^_i_, V^new^_i_, PIDi, h(.)g.

**Figure 5:**
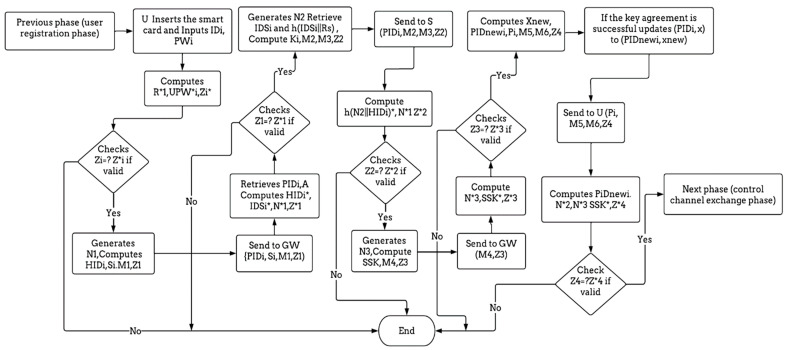
The flow diagram of the login and authentication phase.

After the mutual authentication process is completed successfully, the gateway requests a channel number from the database and sends it to the user and sensor. For further details, Figure 5 summarizes the whole process of the login and authentication phase.

#### 3.3.3. Control Channel Exchange Phase

This stage shows precisely how the control channel is exchanged confidentially and securely after the successful completion of the login and authentication process, as shown in Table 6.

Step 1: This phase begins when the GW sends a request (REQ) for a channel to the DB.

Step 2: The database receives the request and selects an available channel number ch and encrypts it with a KB, which is a pre-shared key between the database and the gateway; it also generates a hash value of this channel {{ch}_KB,_ h(ch)} and sends this message to the GW.

**Figure 6:**
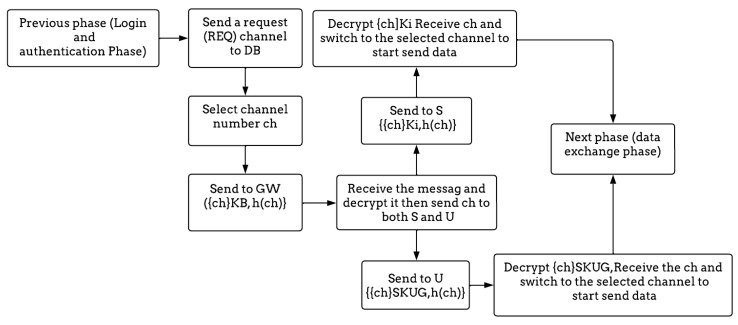
The flow diagram of the control channel exchange phase.

Step 3: The GW receives and decrypts {{ch}_KB_, h(ch)}; then, it sends it to both the sensor and the user. When it sends the channel number to the sensor, it will be encrypted by Ki {{ch}_Ki,_ h(ch)}, but when it sends the channel number to the user, it will be encrypted by SKUG {{ch}_SKUG_, h(ch)}. The sensor decrypts {ch}_Ki_, receives the channel number, and switches to the selected channel to start sending the data. On the other hand, the user also decrypts {ch}_SKUG_, receives the channel number, and switches to the selected channel to start sending data. For further details, Figure 6 summarizes the control channel exchange phase.

#### 3.3.4. Data Exchange Phase

Finally, after exchanging the channel between all parties in the network, they begin to exchange the data securely, as shown in Table 7.

Step 1: After switching to the selected data, the sensor starts sending the data, which are encrypted by SKi, the session key between the sensor and the user {data}_SKi_, and it generates a hash value h(data); then, it sends these to the gateway.

Step 2: The gateway receives and decrypts {{data}_SKi_, h(data)}; then, it determines whether it is valid. After this, the GW forwards the message {{data}_SKi_, h(data)} to the user.

**Figure 7:**
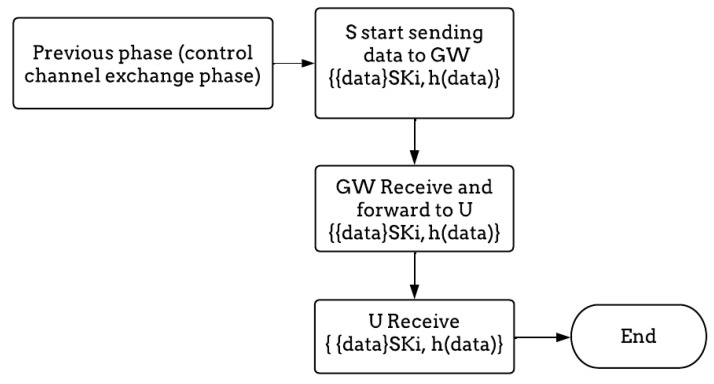
The flow diagram of the data exchange phase.

Step 3: The user receives the data {{data}_SKi_, h(data)} and decrypts them. For further details, Figure 7 summarizes the data exchange phase.

## 4. Security Analysis of the Proposed Protocol Using Ban Logic

In this section, we analyze our proposed protocol, a lightweight authentication MAC protocol for CR-WSNs, by using BAN logic [27], developed by Burrows, Abadi, and Needham to analyze authentication protocols. BAN’s logic depends on believing in the correctness of the other side’s formula. Several variations and improvements have been made to the underlying BAN logic [27,30]. BAN logic is a logic largely dedicated to heuristics and cryptographic protocols. In BAN’s reasoning, the proof procedure is based on the assumptions that constitute effective proof [27,30]. In the lightweight authentication MAC protocol for CR-WSNs, the participants authenticate with each other to establish a session key, SSK, among the U, GW, and S.

### 4.1. Notation and Formulas of BAN Logic

BAN logic consists of principles, encryption keys, and formula statements. The principals are represented by the symbols *P* and *Q*. The encryption keys are represented by K. The formula/statements are represented by the symbols *X* and *Y*, the content of the message exchanged. The logical notations of BAN logic are given below:

- *P* believes *X* holds: P|=X: *P* is enabled to believe *X*: *P* takes the formula *X* as true;
- P≺X: *P* sees /receives the formula *X*: *P* sees the message containing *X* and repeats;
- P |~X: *P* once said the formula *X*: at some time, *P* sent a message including the statement *X*;
- P⇒X: the entity *P* has control over *X* and must be trusted for the formula statement *X;*
- *X* is fresh : #(X), it says, is recent and has not been sent any time before;
- $P\overset{k}{↔}Q$: *P* and *Q* share a secret key (K) only used to communicate between *P* and *Q*. The key is known to *P* and *Q* only and will never be discovered by another party;
- {X}K: the statement encryption of *X* with key K;
- 〈X〉Y: represents the formula *X* combined with *Y*, which means *Y* is a secret that proves the identity of the user who utters 〈X〉Y.
- Table 8 summarizes the basic notations of the BAN logic used in this proof.

### 4.2. Rules

The BAN logic rules are illustrated as follows:

1. Message meaning rules (MMR): $\frac{P|=P\leftarrow^{K}\to Q,P≺\left(X\right)K\;}{P\left|=Q\right|\sim X}$

Message meaning rules concern the analysis of messages. Two of the messages concern the analysis of the encryption, and the third message concerns the analysis of the messages with the secret. All describe how to assume the belief in the original messages. P believes that it shares with Q a secret key; therefore, P sees X encrypted message with key K, and P believes Q sent the message.

2. Nonce verification rule (NVR): $\frac{\;P\left|\equiv \#\left(X\right),P\right|=Q|\sim X}{P\left|=Q\right|=X}$

The nonce verification rule represents the message is recent. The sender believes in it: P believes the fresh message X; P believes Q sent the message. Thus, P believes Q believes X.

3. Freshness rule (FR): $\frac{P|=\#\left(X\right)\;}{P\left|=XP\right|=\left(X,Y\right)}$

If formula one, part known, is fresh, the whole formula needs to be fresh.

4. Believe rule (BR): $\frac{P\left|=Q\right|=\left(X,Y\right)}{P\left|=X,P\right|=Y}$

A necessary property of the belief operator is that the formula P believes a collection of statements, if and only if P believes every individual statement separately.

5. Session key rule (SKR): $\frac{P\left|=Q\#\left(X\right),P\right|=Q|=X}{P|=P\overset{k}{↔}Q}$

A key is used in a communication between the two, P and Q.

### 4.3. Assumptions

The assumptions of the BAN logic in the lightweight authentication MAC protocol for CR-WSNs are described in Table 9.

### 4.4. The Lightweight Authentication MAC Protocol for the CR-WSNs’ Message Exchange

This section illustrates how the messages in our protocol in each phase are applied in BAN logic. Table 10 shows the symbols that are used in the proposed protocol.

#### 4.4.1. Sensor Registration Phase

This phase elucidates the registration process of the entities in the network. Message (1) explains the sensor registration phase. The sensor starts by sending its identity to the gateway as a request to join the network along with the value of the hashes, which are *H*2 and *H*3, so that the gateway can verify the integrity of the sensor’s identity.
*M* (1)S→GW$S|\sim \left\{ID_{Si},\left\{H2,H3\right\}\;S\overset{GSK}{↔}GW\right\}$

$GW⊲\left\{ID_{Si},\left\{H2,H3\right\}\;S\overset{GSK}{↔}GW\;\right\}$

$GW⊲\left\{ID_{Si},\left\{\left\{H2,H3\right\}\;S\overset{GSK}{↔}GW\right\}\;S\overset{GSK}{↔}GW\right\}$

$\left\{Si_{ID},\left\{H2,H3\right\}\;\right\}$

Message (2) shows how the gateway generates the sensor master key, *Ki*, links it with the sensor ID, and sends it to the sensor.
*M* (2) GW→S$GW|\sim \left\{\left\{Ki\right\}\;S\overset{GSK}{↔}GW\;,H4\;\right\}$

$S⊲\left\{\left\{Ki\right\}\;S\overset{GSK}{↔}GW\;,H4\;\right\}$

$S⊲\left\{\left\{\left\{Ki\right\}\;S\overset{GSK}{↔}GW\right\}S\overset{GSK}{↔}GW\;,H4\;\right\}$

{{Ki} ,H4 }

In Message (3), the sensor calculates the session key, *SKi*, which is used to encrypt the data between the sensor and the user, and then sends it to the gateway, which in turn delivers this key to the user when registered.
*M* (3)S→GW$S|\sim \left\{SKi\right\}S\overset{Ki}{↔}GW$

$GW⊲\left\{\left\{SKi\right\}S\overset{Ki}{↔}GW\;\right\}$

$GW⊲\left\{\left\{SKi\right\}S\overset{Ki}{↔}GW\;\right\}S\overset{Ki}{↔}GW$

GW⊲{SKi}

#### 4.4.2. User Registration Phase

Messages (4 to 5) simplify the agreements of the ECC key exchange between the user and the gateway to generate the shared key. Message (6) explains how the gateway, after calculating the derived shared key from the ECC, forwards the calculated session key from the sensor to the user to encrypt the data and send the data securely.

Message (4) contains the user’s *IDi*, the gateway’s *IDG*, the user’s random *RU*, and the certificate’s CUS, signed by the user’s private key, and message (5) contains the user’s *IDi*, the gateway’s *IDG*, the user’s random *RGW*, and the certificate’s *CGS*, signed by the gateway’s private key.

*M* (4)

U→GW



U|~{IDi,IDG,RU,CUS}





GW⊲{IDi,IDG,RU,CUS}





{IDi,IDG,RU,CUS}



*M* (5)

GW→U



GW|~{IDG,IDi,RGW,CGS,PID,HID}





U⊲{IDG,IDi,RGW,CGS,PID,HID}





{IDG,IDi,RGW,CGS,PID,HID}



*M* (6)

GW→U



GW|~{SKi}


$U\overset{SKUG}{↔}GW$



U⊲{{SKi}$U\overset{SKUG}{↔}GW$ }

U⊲{{SKi} $U\overset{SKUG}{↔}GW$ } $U\overset{SKUG}{↔}GW$


U⊲{SKi} $U\overset{SKUG}{↔}GW$

#### 4.4.3. Login and Authentication Phase

This phase describes the mutual authentication between all the entities (*U*, *GW*, and *S*) in the network. Messages (7), (8), (9), and (10) show that every entity should compute some parameters and send them to the next entity, so the next entity can compute the V’s, which are V1, V2, V3, and V4, and validate them. Finally, every entity can authenticate each other.
*M* (7)U→GW$U|\sim \left\{PID_{i},S_{i}\;,M_{1},Z_{i}\right\}$

$GW⊲\left\{PID_{i},S_{i}\;,M_{1},Z_{i}\right\}$

$\left\{PID_{i},S_{i}\;,M_{1},Z_{i}\right\}$

*M* (8)

GW→S    



$GW|\sim \left\{PID_{i},M_{2}\;,M_{3},Z_{2}\right\}$





$S⊲\left\{PID_{i},M_{2}\;,M_{3},Z_{2}\right\}$





$\left\{PID_{i},M_{2}\;,M_{3},Z_{2}\right\}$



*M* (9)

S→GW



$S|\sim \left\{M_{4},Z_{3}\right\}$





$GW⊲\left\{M_{4},Z_{3}\right\}$





$\left\{M_{4},Z_{3}\right\}$



*M* (10) 

GW→U    



$GW|\sim \left\{\;P_{i},M_{5},M_{6},Z_{4}\right\}$





$U⊲\left\{\;P_{i},M_{5},M_{6},Z_{4}\right\}$





$\left\{\;P_{i},M_{5},M_{6},Z_{4}\right\}$



#### 4.4.4. Control Channel Exchange Phase

This phase illustrates the channel exchange between the database, the gateway, the sensor, and the user. It starts when the gateway sends a request for a channel to the database in message (11). In message (12), the database allocates some free channels and sends them to the gateway. In messages (13) and (14), the gateway receives the channel number and sends it to both the sensor and the user with the hash of the channel to ensure the integrity of the message.
*M* (11)GW→DBGW|~{REQ}

DB⊲{REQ}

{REQ}*M* (12)DB→GWDB|~{{ch} $DB\overset{KB}{↔}GW,\;h\left(ch\right)\}$

GW⊲{{ch} $DB\overset{KB}{↔}GW,\;h\left(ch\right)\}$

GW⊲{{{ch} $DB\overset{KB}{↔}GW\}DB\overset{KB}{↔}GW,\;h\left(ch\right)\}$

{ch , h(ch)}*M* (13)GW→SGW|~{{ch} $GW\overset{Ki}{↔}S,\;h\left(ch\right)\}$

S⊲{{ch} $GW\overset{Ki}{↔}S,\;h\left(ch\right)\}$

S⊲{{{ch} $GW\overset{Ki}{↔}S\}GW\overset{Ki}{↔}S,\;h\left(ch\right)\}$

{ch , h(ch)}*M* (14)GW→UGW|~{{ch} $GW\overset{SKUG}{↔}U,\;h\left(ch\right)\}$

U⊲{{ch} $GW\overset{SKUG}{↔}U,\;h\left(ch\right)\}$

U⊲{{{ch} $GW\overset{SKUG}{↔}U\}GW\overset{SKUG}{↔}U,\;h\left(ch\right)\}$

{ch , h(ch)}

#### 4.4.5. Data Exchange Phase

In this phase, the entities switch from the common control channel to the selected channel for secure data exchange. Messages (15) and (16) introduce the data exchange process, where the *S* sends the data to the *GW*; then, the *GW* forwards the message to the *U*. A secure connection is established by defining a private channel for exchanging data and encrypting the data using the session key shared between the user and the sensor node. Additionally, applying the hash value ensures message integrity.
*M* (15) S→GW    S|~{{data}$S\overset{SKi}{↔}U,\;h\left(data\right)\}$

GW⊲{{data}$S\overset{SKi}{↔}GW,\;h\left(data\right)\}$

GW⊲{{{data}$S\overset{SKi}{↔}GW\}S\overset{SKi}{↔}GW,\;h\left(data\right)\}$

{data, h(data)}

*M* (16) 

GW→U    



GW|~{{data}


$S\overset{SKi}{↔}GW,\;h\left(data\right)\}$





U⊲{{data}


$S\overset{SKi}{↔}GW,\;h\left(data\right)\}$





U⊲{{{data}


$S\overset{SKi}{↔}GW\}S\overset{SKi}{↔}GW,\;h\left(data\right)\}$





{data


, h(data)}



### 4.5. Security Analysis of the Proposed Protocol

As we mentioned earlier, the BAN logic methodology is used to determine whether or not the security protocols meet the authentication requirements, so it can be used to define the functions of the proposed protocol to check whether this protocol achieves authentication and also to ensure that the transmission between the sender and the receiver is secure. As a result, by reviewing these areas, we can evaluate the proposed protocol as follows:

- We ensure the integrity of the data using the hash function, as shown in all messages, where the recipient of the message recreates the hash value and compares it with that in the message to ensure that the message has not been tampered with;
- The keys are generated using the elliptical curve algorithm in the registration phase of the user, where the elliptical curve factors are first exchanged between the two ends in order to generate the shared key for both terminals, as shown in messages (4) to (5) in the user registration phase; in message (6), the gateway sends the session key to the user for use in the data exchange phase. The shared key is generated between the two parties. The elliptic curve algorithm has many characteristics, including being a powerful algorithm that is exceedingly difficult to crack, with a small key size, fast performance, and limited power consumption;
- Messages (7), (8), (9), and (10) achieve mutual authentication, so when any node user is registered and authorized to join the network, they become legitimate users once they own a shared key generated by an elliptical curve that is shared by the gateway and other network entities separately. Therefore, each node is able to easily authenticate with each other at the login and authentication stage. Thus, participants authenticate with each other to generate an *SK*, session key, between the *U*, the *GW*, and the *SN*.
- After the authenticated process is completed successfully, in messages (11) to (14), the gateway sends a request to the database asking for a free channel, whereas the ch is sent encrypted so as to be unrecognized by the hacker in order to prevent this channel being busy. Moreover, it is sent with the hash to ensure the channel’s validity.
- Messages (15) and (16) allocate a channel for transmitting data through the gateway, the purpose of which is to ensure secure communication between the sensor nodes and the user. Therefore, confidentiality is ensured when the data are encrypted by the session key, and no one else besides the sender and the receiver who have the keys can decrypt the data and know the content of the message without the key.
- The authentication process requires each entity to be registered in messages (1 to 3) and (4 to 6) in the user registration phase in the network, when the sensor or the user requested a channel that required the GW to check the validity of that user based on the hashes and the keys between them. So, it is difficult for the hacker to pretend to be a legitimate user, which leads to detecting the hacker immediately.

## 5. The Implementation and Evaluation of the Proposed Lightweight Authentication MAC Protocol for CR-WSNs

The lightweight authentication MAC protocol for the CR-WSN not only relies on lightweight features using XOR operations and hash functions but also ensures a remarkably high level of security compared to the related systems due to the use of the elliptical curve algorithm in one phase of the proposed protocol. To assess the security of the lightweight MAC authentication protocol for the CR-WSN networks, we performed informal security analysis and formal security analysis, such as BAN logic and the AVISPA simulation tool. We show that the proposed protocol prevented a variety of attacks using informal analysis. In this section, we demonstrate the MAC lightweight authentication mutual protocol for CR-WSNs using BAN logic and use the AVISPA Emulator to demonstrate the MAC lightweight authentication protocol for the security features of the CR-WSNs against replay attacks and MITM attacks.

### 5.1. Informal Security Analysis

This subsection is concerned with explaining the security of the proposed protocol against many types of attacks, for instance, sensor node capture, replay, stolen user smart card, insider, privileged insider, MITM attacks, and other attacks. Furthermore, the lightweight authentication MAC protocol for CR-WSN ensures mutual authentication and perfect forward secrecy.

#### 5.1.1. Insider Attack

Let us assume A is an attacker. We suppose A was able to register on the network as a legitimate user. A managed to enter the authentication phase with the gateway and the sensor and gains these messages from the S and the GW, for instance, {PIDi, M2, M3, Z2} from the GW, {M4, Z3} from the sensor, and {Pi, M5, M6, Z4} also from the GW. Therefore, A wants to calculate the session key, so A needs to compute all the nonce. However, if A tries to compute h (Ki ∥ PIDi) =M2 ⨁ h (N2 ∥ HIDi) or h (h (N2 ∥ HIDi) ∥ Ki) = N1 ⨁ M3, in both cases, A cannot because A does not know the shared secret key, Ki, between the S and the GW. Therefore, the lightweight authentication MAC protocol for the CR-WSNs is immune to this attack.

#### 5.1.2. Stolen Smart Card Attack

Every legitimate user in the network has a smart card. In this attack, A tries to steal the user’s smart card and analyze the smart card to extract the stored secret parameters in the card. Then, A tries to complete the authentication process with both the GW and the S, but A cannot send the login message, which is {PIDi, Si, M1Z1}, because A needs to calculate HIDi, and to do so, A needs to guess both the PWi and the IDi at the same exactly time. So, since it is exceedingly difficult to obtain them both at the same exact time, the proposed protocol is immune and resistant to this type of attack.

#### 5.1.3. Replay Attack

In the lightweight authentication MAC protocol for the CR-WSN networks, there are four stages. We suppose that in the login and authentication phase, A tries to capture some messages from a legitimate user, such as {PIDi, Si, M1, Z1} or {PIDi, M2, M3, Z2}. A wants to start the authentication process with the GW in new sessions by using the captured messages. A can never do that because the sensor nodes ensure all random numbers, such as N1, N2, and N3, are up to date. Therefore, we can say that the proposed protocol is very secure against replay attacks.

#### 5.1.4. Sensor Node Capture Attack

In this scenario, even if A could obtain a certain sensor and extract information such as Si_ID_ or Ki from its memory, A could not threaten any other sensor node in the network. A can only perform authentication with the gateway and the user using this node because each sensor has its own key associated with its identity Ki = h (h (Si, ID, Rs) || GWS). Therefore, A cannot threaten or collect any other information about other sensors in the network. The network is safe and impervious to this attack.

#### 5.1.5. Offline Password Guessing Attack

Let us assume that attacker A was able to guess the secret password of a legitimate user in the network. A was also able to know all the information stored in the smart card of the legal user {SRi, UHIDi, Vi, PIDi, K} and then pretends to be the legitimate user inside the network using this information. In order to be able to calculate this message in the authentication stage {PIDi, M2, M3, Z2}, A must know the HID, and A cannot calculate the HID without the Ri account first; A will not be able to calculate it because A does not have the identity of this legal user. Therefore, the proposed protocol is protected against this attack.

#### 5.1.6. Mutual Authentication

In the lightweight authentication MAC protocol for the CR-WSN, we accomplish mutual authentication. The mutual authentication process depends on validating some parameters. The validation process is based on complex multiple sequential hashes generated based on the secret information provided; therefore, it is too hard to predict them. Thus, every entity in the network authenticates with each other by performing verification processes. First, the gateway audits the validation of Z1 = ? Z1. Then, the sensor node audits the validation of Z2 = ? Z2. After that, it returns to the GW to inquire about the validation of Z3 = ? Z3. Finally, the user audits the validation of Z4 = ? Z4. Eventually, if the entire process is completed successfully, we can say that the proposed protocol fulfills mutual authentication.

#### 5.1.7. Privileged Insider Attack

In this attack, the scenario is as follows: If there is an internal adversary (A) with privilege, and A is able to capture and know the ID of the legitimate user, then A will try to find out the shared session key, SK = h(h(N2||HID) || N3||N1), by looking at the messages at the authentication stage. In order to calculate this key, A needs to know the HID, which is the secret shared parameter between both the user and the gateway. A cannot calculate the HID either from the identity of the legitimate user or from this message {PIDi, Si, M1, Z1} without knowing the random number and password of this user; so our proposed protocol ensures protection against this type of attack.

#### 5.1.8. Stolen Verifier Attack

Suppose A steals the validation table of the gateway inside the network and has the sensor ID as well as the value of the Si, ID, H2, and (PIDi, A). A, with all this information, cannot calculate the session key, SK = h(h(N2||HID) || N3||N1), because A does not have an HID, and even if A tries to calculate the HID using PIDi = HIDi ⨁ h(A ∥ GWS), A will not be able to have the GWS, which is the secret parameter of the gateway; so, the proposed protocol is completely secure against this attack.

#### 5.1.9. MITM Attack

The man-in-the-middle attack is a common attack, which mostly occurs in the second phase, the login and authentication phase. It is possible for the attacker to intercept and capture messages, such as capturing the message {PIDi, Si, M1, Z1}, modify it, and tamper with it. Still, the gateway can quickly detect this type of attack by looking in the local database. Moreover, it is worth noting that if some messages are modified, it is impossible to change them all. Thus, we ensure that the proposed protocol is secure against the man-in-the-middle attack.

#### 5.1.10. Session-Specific Random Number Leakage Attack

A session random number leak attack completely depends on leaking all or some random parameters of the login and authentication stage. Even if A knows this, A will then try to calculate the session key, SK = h(h(N2||HID) || N3||N1). Our proposed protocol guarantees that A cannot calculate this key without knowing the HID, whose knowledge depends on knowing the secret parameter of the GSW gate or its random number.

#### 5.1.11. Perfect Forward Secrecy

In this attack, even if A can find and know the secret parameter of the gateway, A can only calculate the shared session key by knowing one of two: h(A ∥ GWS) or h(h (Si, ID, Rs) || GWS). So, A has to obtain A or h (Si, ID, Rs) to analyze the GSW. The lightweight authentication MAC protocol for CR-WSN guarantees complete secrecy forward.

### 5.2. Formal Security Verification Using the AVISPA Simulation

The AVISPA simulation tool stands for the Automated Validation of Internet Security Protocols and Applications. This tool is usually used to simulate the security of the authentication protocols and verify them [30,31,32]. High-Level Protocol Specification Language (HLPSL) is a powerful language used by the AVISPA tool, thus allowing us to verify the protocol security features [33].

**Figure 8:**
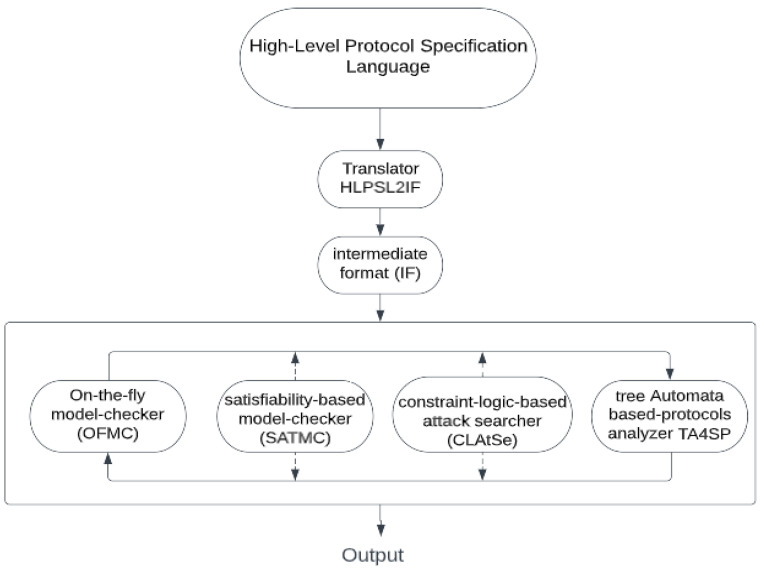
The AVISPA simulation tool architecture.

Figure 8 shows the AVISPA tool architecture. To start, a text is written in CAS+ language and given as input to SPAN, which in turn will convert it to an HLPSL text [34]. Then, this text is entered into the intermediate format (IF) translator, which in turn directly converts it to an intermediate format (IF) by using the translator HLPSL2IF [34]. Then, it analyzes it by using one of four backends existing in the AVISPA tool: the on-the-fly model-checker (OFMC), the constraint-logic-based attack searcher (CLAtSe), the satisfiability-based model-checker (SATMC), and the tree automata based on automatic approximations for the analysis of security protocols (TA4SP) [34,35]. One of the selected backends executes the protocol during a number of iterations until it determines that the protocol is secure or an assault is found, in order to check all the targets previously defined in the HLPSL targets section. The AVISPA tool is very flexible because it enables the user to use it in two diverse ways; the first is through the AVISPA security protocol animator (SPAN), a program with a very easy-to-use graphical interface simulating the encryption protocol. The second method is through calling the default command-line options of the automated validation of the internet security protocols tool [34]. Specifically, the first method SPAN was adopted with the OFMC backend because it contained XOR operations in the proposed protocol.

#### 5.2.1. HLPSL Specification

The HLPSL contains three specifications. First, the roles must be defined. The roles are divided into two parts; the first part comprises the main roles played by agents, and the second part comprises the configuration roles, which show what needs to be considered during the situation in analyzing the protocol. Second, we define the objectives that the proposed protocol must meet. The third is to instantiate the main role with the arguments passed [34]. The main roles in the proposed protocol are shown in Appendix A and defined as follows: the user obtains the sensed data from the gateway that have been collected from the sensors. The gateway is responsible for the key generation, for pre-requesting the channels from the DB, and for allocating the channels to the sensors and the users, as well as completing the authenticating process mutually, where every entity in the network can authenticate each other. The sensor obtains some secret parameters for authentication and the collection of data. The database is an entity that provides a list of available TV channels. The main role’s task is to explain the initial knowledge of all the participating protocol entities, such as the initial state and all the transitions that occur between these participating entities [34]. The Doley–Yao threat model [36] implemented by the HLPSL validates a reboot and man-in-the-middle attack. This model demonstrates that the attacker has the ability to capture and compose any message as well as eavesdrop.

#### 5.2.2. Analysis of the Simulation Results

**Figure 9:**
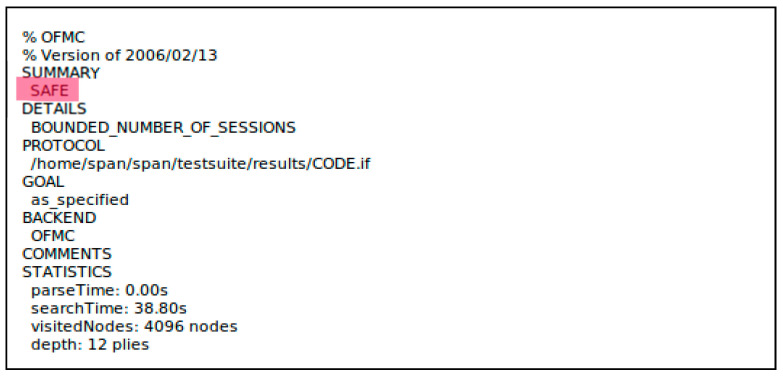
The results of the AVISPA simulation of the lightweight authentication MAC protocol for the CR-WSNs.

The simulation of our proposed protocol was conducted by the AVISPA tool simulator, and it was used for testing the security aspect related to the reply attack and the man-in-the-middle attack in the lightweight authentication MAC protocol for the CR-WSNs. The results of simulating the proposed protocol are shown in Figure 9; the results indicate that the protocol is SAFE under the OFMC backend simulation, which utilized the XOR operations that were adopted in the proposed protocol. This means that the proposed protocol is secure and immune against man-in-the-middle and replay attacks. Figure 9 also shows the total search time = 38.80 s, the number of visited nodes = 4096 nodes, and the depth = 12 plies. The proposed lightweight authentication MAC protocol for the CR-WSNs was secure against all passive and active attacks.

### 5.3. Performance Analysis

This section explains how to calculate the computational costs and security features of the proposed lightweight authentication MAC protocol for the CR-WSNs and then compares them with the related protocols [4,21,37,38,39].

#### 5.3.1. Computational Costs

Based on the performance of the related current schemes [4,21,37,38,39], we analyzed the computational cost of the proposed protocol, using a computer with a quad-core CPU of 3.2 GHz with 8 GB memory. We considered the following estimate, with T_h_ as the execution time of the hash function (0.00032 s), the T_ecm_ double ECC score (0.0171 s), and finally the T_sym_ symmetric encoder/decryption (0.0056 s). We did not consider the XOR execution time because it was negligible. Table 11 shows the results of the computational costs.

**Figure 10:**
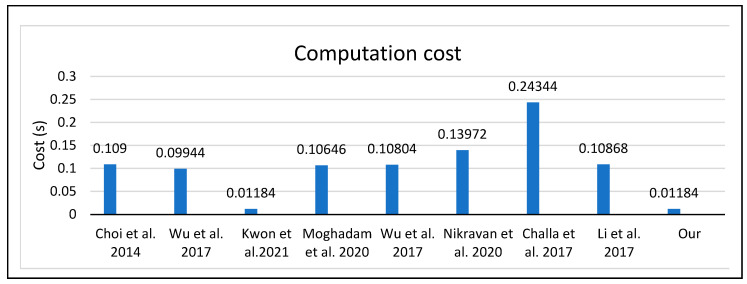
Total computational cost comparison [4,21,37,38,39,40,41,42].

Table 11 and Figure 10 demonstrate the computational cost comparison of the proposed and some related protocols. The computational cost was calculated at the login and authentication stage. The total cost of the proposed protocol equals the total cost delivered, as defined by the research work presented in [4], which was equal to 0.01184 at the login and authentication phase; the reason for this, as shown in Table 11, was that the two protocols were equal in the number of hashes, 37T_h_, but the operations in the proposed protocol differed completely in several other stages from [4], for example, the sensor registration phase, the user registration phase, the control channel exchange phase, and the data exchange phase.

The two protocols were also completely different since the proposed protocol operates in the cognitive radio-based wireless sensor network, while the protocol presented in [4] was limited to the WSN environment. The other phases of the proposed protocol were also completely different, including the process of the registration phase of the proposed protocol, since it was based on the ECC algorithm; further, the multiple symmetric keys involved increasing the level of security within the registration process, while the process of the registration phase of the research work [4] considered only the secure channels’ assumptions. In addition, the control channel exchange phase only existed in the proposed protocol, since it was an essential part of the cognitive entities to exchange their available channels for data transmissions.

On other hand, the other total cost of the protocols shown in Table 11 was higher than the proposed protocol due to the number of security algorithms that were used within the login and authentication phase; for example, in [41], they required high computation compared to recent schemes because they used a signature-based secure certification key generation system for IoT applications, while the following papers [37,38,39,40,42] showed almost identical results. Moreover, paper [21] used ECC, encryption, and decryption in the login and authentication process, thus obtaining a higher cost value than the cost of the proposed protocol. Therefore, the proposed protocol outperformed all these related protocols and is suitable for the CR-WSN environment.

#### 5.3.2. Security Properties

Table 12 compares the proposed protocol to the related protocols [4,21,37,38,39,40,41,42] in terms of the security characteristics. The table clearly shows that all these protocols suffered from many attacks, while the proposed protocol addressed all of them, which confirmed its safety.

## 6. Conclusions and Future Work

While wireless network technology, known for its wide applications in most areas, may have some security challenges and issues that need to be considered to fix and improve the network, our extensive research led to the conclusion that wireless networks mainly suffer from a shortage of spectrum, which is caused by the unreasonable demand for use by the users. On the other hand, CRNs use the unused spectrum in a very promising way. Researchers are beginning to explore this. Thus, to increase the performance and security of wireless networks, it may be possible to implement CR technology that integrates with adaptive systems to improve their performance and security in a significant and efficient way. Many aspects of security associated with both WSNs and CRNs, particularly the authentication aspect of security, must be addressed so that WSNs can be better implemented and secured. In the context of the CR-WSN, the MAC protocol was proposed as a lightweight authentication method, and with the support of BAN logic, the proposed protocol was analyzed. To provide formal verification, AVISPA was used. In sum, the result clearly exhibited that the lightweight authentication MAC protocol for CR-WSNs is safe under the OFMC backend.

In future work on the proposed protocol, CR-WSNs will be fully simulated to create an environment suitable for testing the proposed protocol to verify its functionality and performance. The proposed protocol will be simulated with 100 nodes to measure the network performance, connection time, and computational time when users and sensors switch to the selected channel for secure data exchange.

## Figures and Tables

**Table 1:** Comparison of the most recent relevant approaches.

Reference	Year	Approach	Security Algorithms	Network	Result
[22]	2017	A two-level authentication system was proposed to verify the authenticity of the cognitive node and its user.	symmetric-key cryptography and public-key infrastructure-based	CRNs	The result decreased the authentication time needed to accomplish the number of cryptographic operations and the authentication process.
[23]	2010	A trust-based authentication mechanism for secure communication in CRNs was proposed.	trust-based authentication mechanism	CRNs	This secure authentication reduced the relative calculation overheads and communication costs.
[18]	2020	A lightweight privacy-preserving scheme for secondary users in database-driven CRNs was proposed.	lightweight operations such as XOR and hash function	CRNs	Their results showed that their system outperformed previous schemes in terms of the communication cost and computation and incurred the shortest delay in channel allocation.
[19]	2020	A secure lightweight three-factor-based user authentication protocol was proposed for WSNs.	hash and XOR operations	WSNs	Their scheme provided good and efficient communication and computation costs related to other existing schemes.
[21]	2020	A key agreement mechanism and an ECDH-based authentication were proposed for the WSN infrastructure.	key agreement and mutual authentication protocol based on the elliptic curve Diffie–Hellman	WSNs	It supported more security attributes, and the proposed protocol performance was more effective than other related protocols.
[25]	2020	For providing security in the CRSN, the authors in this paper performed an elliptic curve digital signature algorithm-based spectrum aware cryptography.	elliptic curve digital signature algorithm (ECDSA)	CR-WSNs	The simulation findings showed that the ECDSA-based SAC algorithm dramatically reduced packet latency and energy usage.
[26]	2020	Collaborative spectrum secure (CSS) sensing technology based on the mechanism’s reputation for wireless cognitive sensor networks was proposed	Collaborative spectrum secure (CSS) sensing based on reputation	CR-WSNs	The results showed the proposed method outperformed previous methods in terms of sensing performance and excelled at detecting and isolating attackers.
[4]	2021	A secure and lightweight mutual authentication protocol for WSN-SLAP was proposed.	hash functions and XOR operations	WSNs	The approach provided perfect forward secrecy and mutual authentication.

**Table 2:** Notations of the proposed lightweight authentication MAC protocol for CR-WSNs.

Notations	Description
GW	Gateway
Uj	User
Si	Sensor
DB	Database
ID_Si_	Sensor’s ID
ID_i_	User’s ID
ID_G_	Gateway’s ID
GSK	Pre-shared key between gateway and sensor
GWS	Secret parameter of the GW
Ki	Sensor’s master key
SKi	Sensor’s session key
Upk	User’s private key
Upub	User’s public key
Q	A base point of order n over Eq
PW_i_	Password of user
PID_i_	Pseudo identity of user
GWpk	Gateway’s private key
GWpub	Gateway’s public key
SKUG	Shared key between gateway and user
N1, N2, N3	Random nonce
Rs, RUU, A, R_g_, RGG, Ri	Random numbers
h(.)	Hash function
||	Concatenation function
⊕	Exclusive-or function

**Table 3:** Sensor node registration phase.

Sensor S_i_	Gateway GW
Retrieve GSK, the pre-shared key between gateway and sensor Generate a random number Rs Select ID_Si_ Generate a hash H1 = h(ID_Si_) Generate a hash H2 = h (ID_Si_, Rs) Generate a hash H3 = H1 ⊕ H2	
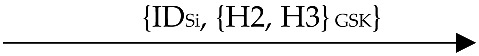	GWS, the secret information of the GW Decrypt {ID_Si_, {{H2, H3} _GSK_} ^GSK^} Generate a hash H*1 = h(ID_Si_), compute H*3 = H*1 ⊕ H2 Compare H3 = ? H*3. If valid Generate sensor master key Ki = h (h (ID_Si_, Rs) || GWS) Generate a hash H4 = h (Ki || ID_Si_) ⊕ H2 Then, link ID_Si_ with Ki sensor master key and store {ID_Si_, H2, Ki} in the local database
Compute SKi = h (H2 || Ki) ⊕ h (Rs) 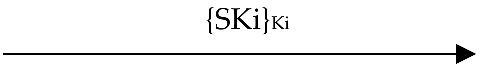	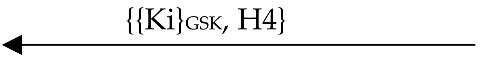 Link SKi with ID_Si_ and send a copy to the user

**Table 4:** User registration phase.

User U_j_	Gateway GW
Select Upk, compute Upub = Upk * Q Generate a random number RUU, R_i_ Compute HU = h (RUU || Upk), compute RU = HU * Q Compute KU = Upk * ID_G_, compute CUS = h (IDi || ID_G_ || RU || KU)	
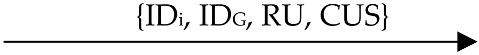	GWS is the secret information of the gateway Generate a random number A, R_g_ Compute HID_i_ = h (ID_i_ ∥ R_g_) and PIDi = HIDi ⊕ h(A ∥ GWS) Select GWpk, compute GWpub = GWpk * Q Generate a random number RGG Compute HG = h (RGG || GWpk), compute RGW = HG * Q, Compute KG = GWpk * IDi, compute CGS = h(ID_G_ || IDi || RGW || KG) 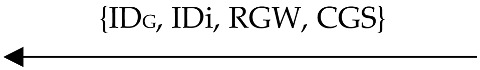
Compute K = HU * RGW Compute SKUG = h (IDi || ID_G_ || RU || RGW|| K), compute UPWi = h (PWi ∥ Ri) SRi = Ri ⊕(IDi ∥ PWi) UHIDi = HIDi ⊕ h (PWi ∥ IDi ∥ Ri) Zi = h (UPWi ∥ IDi ∥ Ri) Store {SRi, UHIDi, Vi, PIDi, K, h(.)} In the smart card	Compute K = HG *RU Compute SKUG = h (IDi || ID_G_ || RU || RGW|| K) Forward the session key that was calculated from the sensor node Ski Store {PID, HID, A} in the database 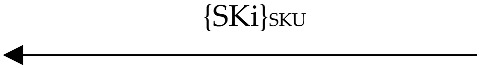

**Table 5:** Login and authentication phase.

User U_j_	Gateway GW	Sensor S_i_
Insert the smart card Input IDi, PWi Compute R* _1_ = SRi ⊕ h(IDi ∥ PWi) UPW*i = h(PWi ∥ Ri) Zi* = h(UPWi ∥ IDi ∥ R*_1_) Check Z*_i_ =? Z_i_ Generate a random nonce N1 Compute HID_i_= UHIDi ⊕ h(PWi ∥ IDi ∥ Ri) Si = ID_Si_ ⊕ h(PIDi ∥ HIDi) M1 = N1 ⊕ h(HIDi ∥ PIDi) Z_1_ = h(ID_Si_∥ PIDi ∥ N1 ∥ HIDi) 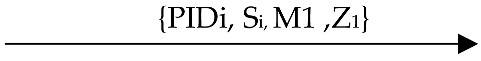		
	Retrieve PIDi and the secret value A Compute HID*_i_ = PIDi ⊕ h(A ∥ GWS) IDSi *= Si ⊕ h(PIDi∥ HID*_i_) N*_1_ = M1 ⊕ h(HID*_i_∥ PIDi) Z_1_* = h(ID_Si_*∥ PIDi ∥ N*_1_ ∥ HID*i) Check Z *_1_ =? Z_1_ Generate a random nonce N2 Retrieve ID_Si_ and h(ID_Si_ ∥ Rs) Compute Ki = h(H2 || KGW) M2 = h(N2 ∥ HIDi) ⊕ h(Ki ∥ PIDi) M3 = N1 ⊕ h(h(N2 ∥HIDi) ∥Ki) Z_2_ = h(PIDi∥ ID_Si_ ||h(N2||HIDi)||N1)	
	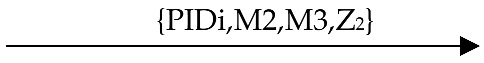	Compute h(N2 ∥ HIDi)* = M2⊕ h(Ki ∥ PIDi) N1* = M3 ⊕ h(h(N2 ∥HIDi) ∥Ki) Z_2_* = h(PID|| ID_Si_ ||h(N2||HID)||N1* Check Z_2_ = Z_2_* ?? Generate a random nonce N3 Compute SSK = h(h(N2||HID)||N3||N1) M4 = N3 ⊕ h(Ki ||N2) Z3 = h(SSK||N3|| ID_Si_)
	Compute N3* = M4 ⊕ h (Ki ||N2) SSK* = h(h(N2||HID) ||N3*||N1) Z_3_* = h (SSK*||N3*|| ID_Si_) Check Z_3_ = Z_3_*?? Compute x^new^ = h (A || N2) PID^new^ i = HIDi ⊕ h (x^new^ || KGW) Pi = PID^new^i ⊕ h (N1 || HIDi) M5 = N2⊕ h (HID|| ID_Si_ ||N1) M6 = N3 ⊕ h(N2||HID||PIDnew) Z_4_ = h(N2||N3||PIDnew||SSK) If the key agreement is successful, update {PIDi, x} to {PID ^new^i, xnew}	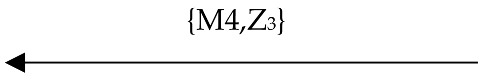
Compute PID ^new^i = Pi ⊕ h(N3||HIDi) N2* = M5 ⊕ h (HID|| ID_Si_ ||N1) N3* = M6 ⊕ h(N2||HID||PIDnew) SSK* = h(h(N2||HID) ||N3*||N1) Z_4_* = h(N2||N3||PIDnew||SSK) Check Z_4_ = Z_4_*??	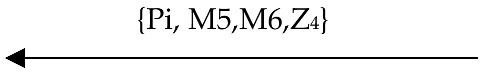	

**Table 6:** Control channel exchange phase.

Database DB	Gateway GW	Sensor S_i_	User U_j_
	Send a request (REQ) channel to DB		
Select channel number ch	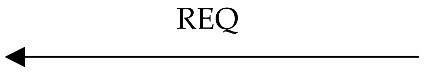		
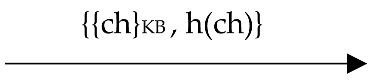	Receive ch and decrypt it; then, send it to both sensor and user		
	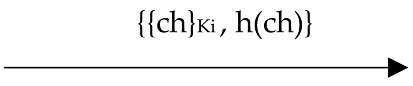	Decrypt {ch}_Ki_ Receive the channel number and switch to the selected channel to start send data	
	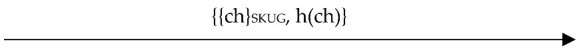		Decrypt {ch}_SKUG_ Receive the channel number and switch to the selected channel to start send data

**Table 7:** Data exchange phase.

Sensor S_i_	Gateway GW	User U_j_
Start sending data		
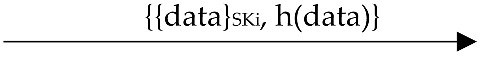	Receive and forward {{data}_SKi_, h(data)}	
	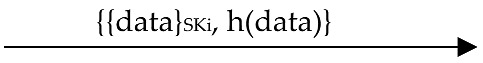	Receive the data {{data}_SKi_, h(data)}

**Table 8:** Notations of BAN logic adopted in the lightweight authentication MAC protocol for the CR-WSNs.

Notations	Description
P1, P2	Two principals
S1, S2	Two statements
SK	The session key
P| ≡ Q	P believes Q
P|~ Q	P once said Q
P| ⇒ Q	P1 controls Q
P ⊲ X	P1 receives Q
#S1	S1 is fresh
{S1} Key	S1 is encrypted with key
P1↔P2	P1 and P2 have a shared key
K^−1^	Private key

**Table 9:** Assumptions used in the lightweight authentication MAC protocol for the CR-WSNs.

Assumptions	Explanation
$S|\equiv S\overset{GSK}{↔}GW$	The S believes the S has a shared key with the *GW*
$GW|\equiv S\overset{GSK}{↔}GW$	The GW believes the S has a shared key with the *GW*
$S|\equiv S\overset{Ki}{↔}GW$	The S believes the S has a shared key with the *GW*
$GW|\equiv S\overset{Ki}{↔}GW$	The GW believes the S has a shared key with the *GW*
$S|\equiv S\overset{SKi}{↔}GW$	The S believes the S has a shared key with the *GW*
$GW|\equiv S\overset{SKi}{↔}GW$	The *GW* believes the *S* has a shared key with the *GW*
$DB|\equiv DB\overset{KB}{↔}GW$	The *DB* believes the *DB* has a shared key with the *GW*
$GW|\equiv DB\overset{KB}{↔}GW$	The *GW* believes the *DB* has a shared key with the *GW*
$GW\left|\equiv U\right|\sim |\overset{GW_{pub}}{\to }GW$	The gateway believes the *U*, if only the user is signed and has been given the gateway’s public key
$U\left|\equiv GW\right|\Rightarrow |\overset{GW_{pub}}{\to }S$	The *U* believes the gateway once it is registered and has been given the gateway’s public key
$GW\left|\equiv \right|\overset{U_{pub}}{\to }U$	The gateway believes the public key of the user

**Table 10:** The variables that are used in the lightweight authentication MAC protocol for the CR-WSNs.

Symbol	Definition
S, U, GW, DB	Principals
GSK	Shared key between the sensor and the gateway
Ki	Sensor’s master key
SKi	Session key
$\overset{GW_{pub}}{\to }\;GW$	Gateway public key
$\overset{GW_{PK^{-1}}}{\to }GW$	Gateway private key
$\overset{U_{pub}}{\to }\;U$	User’s public key
$\overset{U_{PK^{-1}}}{\to }U$	User’s private key
$U\overset{SKUG}{↔}GW$	Shared key between the user and the gateway
ECC-P	Elliptic curve parameter
KB	Shared key between the database and the gateway

**Table 11:** Comparison of the login and authentication computational costs.

Schemes	Gateway	User	Sensor	Total	Total Cost (s)
Choi et al. [37]	6T_h_ + 2T_ecm_	9T_h_ + 3T_ecm_	5T_h_ + 1T_ecm_	20T_h_ + 6T_ecm_	0.109
Wu et al. [38]	11T_h_ + 2T_sym_	12T_h_ + 2T_ecm_ + 1T_sym_	4T_h_ + 2T_ecm_ + 1T_sym_	27T_h_ + 4T_ecm_ + 4T_sym_	0.09944
Kwon et al. [4]	18T_h_	13T_h_	6T_h_	37T_h_	0.01184
Moghadam et al. [21]	1T_ECC_ + 5T_h_ + 2T_sym_	6T_h_ + 3T_ECC_ + 1T_sym_	1T_ECC_ + 2T_h_	13T_h_ + 5T_ECC_ + 3T_sym_	0.10646
Wu et al. [39]	8T_h_ + T_ECC_	5T_h_ + 3T_ECC_	4T_h_ + 2T_ECC_	17T_h_ + 6T_ECC_	0.10804
Nikravan et al. [40]	5T_h_ + 4T_ECC_ + 2T_sym_	5T_h_ + T_ECC_ + 2T_sym_	1T_h_ + 1T_ECC_ + 2T_sym_	11T_h_ + 6T_ECC_ + 6T_sym_	0.13972
Challa et al. [41]	5T_ECC_ + 4T_h_	5T_ECC_ + 5^Th^	4T_ECC_ + 3T_h_	14T_ECC_ + 12T_h_	0.24344
Li et al. [42]	7T_h_ + T_ECC_	8T_h_ + 3T_ECC_	4T_h_ + 2T_ECC_	19T_h_ + 6T_ECC_	0.10868
Ours	18T_h_	13T_h_	6T_h_	37T_h_	0.01184

**Table 12:** Security properties.

Security Property	[4]	[37]	[21]	[38]	[39]	[40]	[41]	[42]	Ours
MITM Attack	✓	✓	✓	✓	×	✓	✓	×	✓
Session-Specific Random Number Leakage Attack	✓	×	×	×	×	×	×	×	✓
Privileged Insider Attack	✓	✓	✓	✓	×	×	×	×	✓
Stolen Smart Card Attack	✓	×	✓	×	×	✓	✓	✓	✓
Perfect Forward Secrecy	✓	✓	×	✓	✓	✓	✓	✓	✓
Replay Attack	✓	✓	✓	✓	✓	✓	✓	✓	✓
Sensor Node Capture Attack	✓	✓	✓	✓	✓	×	✓	×	✓
Offline Password Guessing Attack	✓	×	✓	×	✓	✓	✓	×	✓
Key Compromise Impersonation	×	×	×	×	×	×	×	×	✓
Mutual Authentication	✓	✓	✓	✓	✓	✓	✓	×	✓
Stolen Verifier Attack	✓	×	✓	✓	✓	×	×	×	✓
Insider Attack	✓	✓	×	✓	×	×	✓	×	✓

**Figure A1:**
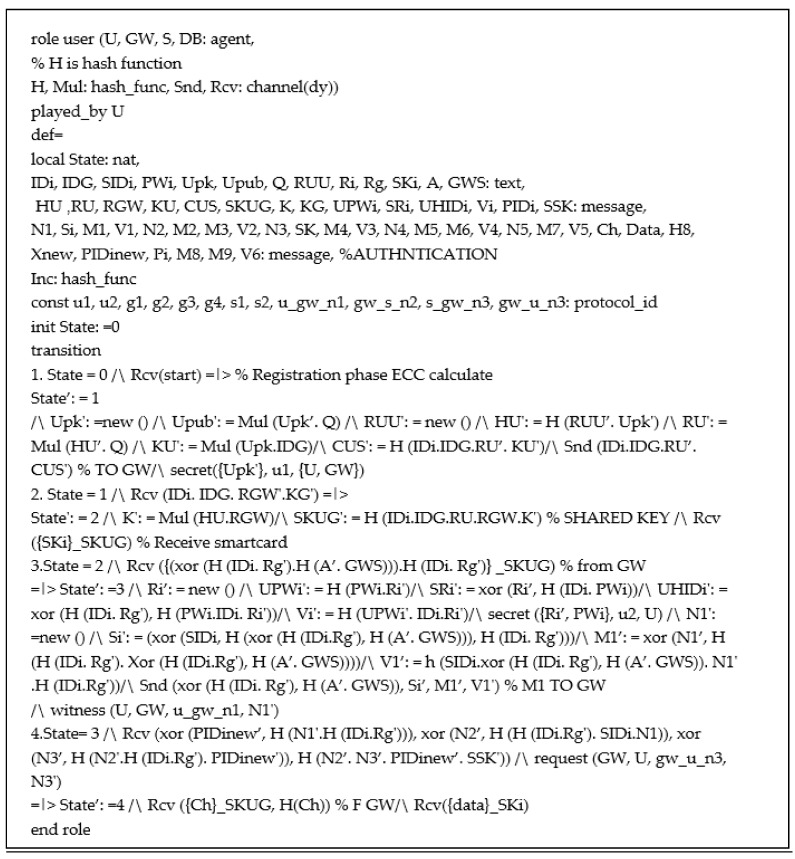
Role of the user.

**Figure A2:**
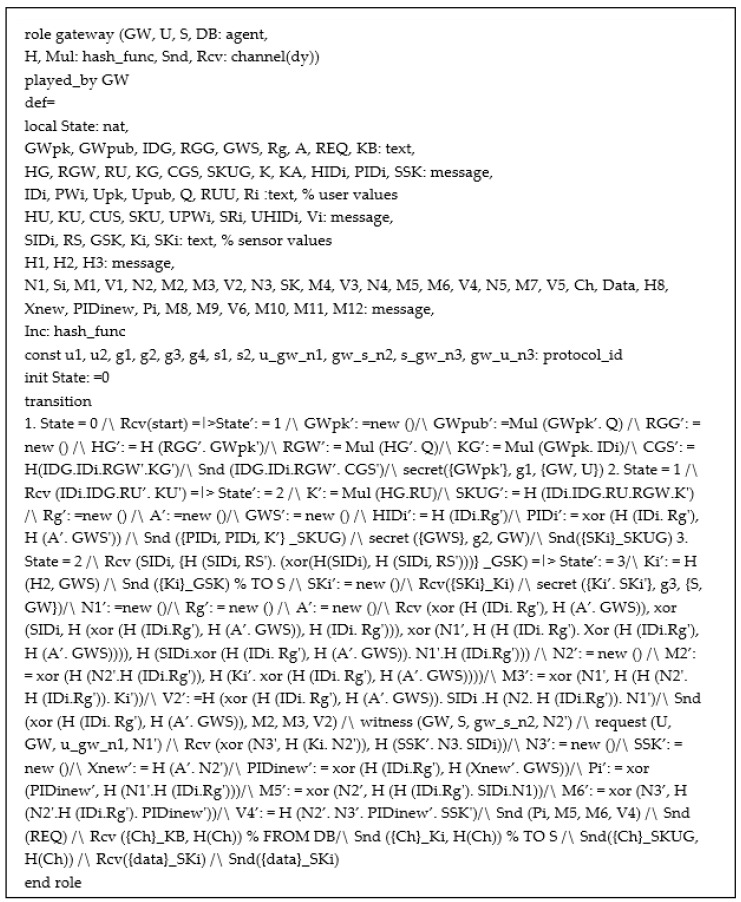
Role of the gateway.

**Figure A3:**
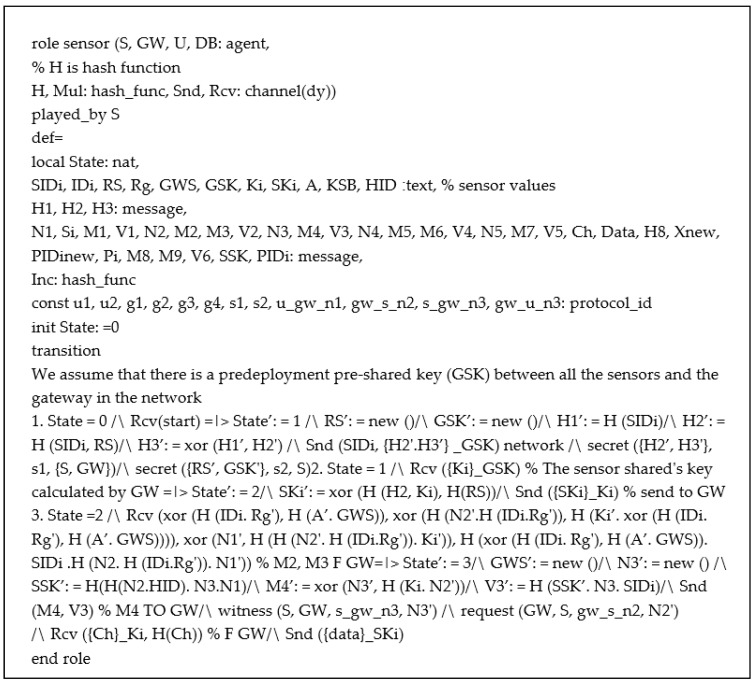
Role of the sensor.

**Figure A4:**
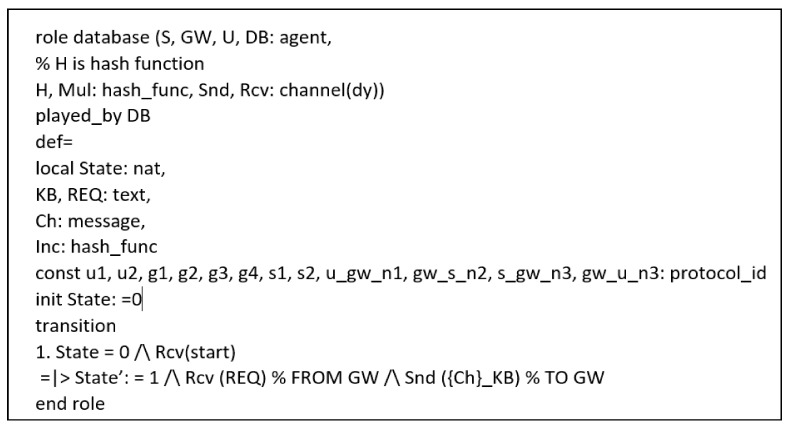
Role of the database.

**Figure A5:**
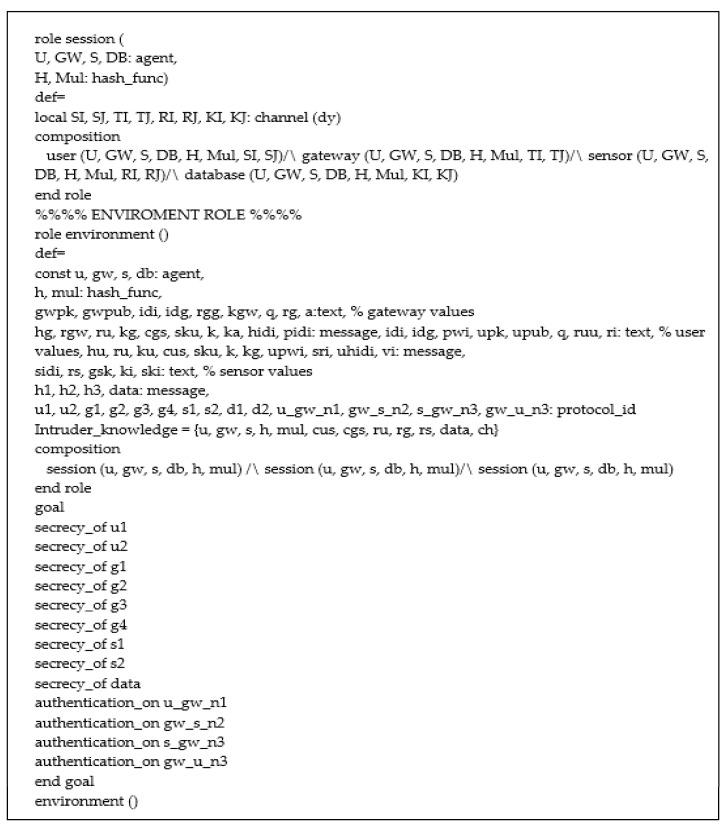
Role of the session and environment.

## Data Availability

Not applicable.

## Appendix A

Figure A1 illustrates the role of user through four states as shown in the following figure.

Figure A2 illustrates the role of gateway through three states as shown in the following figures.

Figure A3 shows the role of sensor through three states as shown in the following figure.

Figure A4 demonstrates the role of database through one0 state as shown in the following figure.

Figure A5 illustrates the role of session and the environment as shown in the following figure.
